# The Relationship Between Compulsive Buying and Hoarding in China: A Multicenter Study

**DOI:** 10.3389/fpsyg.2021.721633

**Published:** 2021-10-15

**Authors:** Heping He, Meihua Zhu, Simon Ching Lam

**Affiliations:** ^1^College of Management, Shenzhen University, Shenzhen, China; ^2^School of Nursing, Tung Wah College, Kowloon, Hong Kong, SAR China

**Keywords:** compulsive buying, compulsive hoarding, common method bias, Hong Kong, mainland China

## Abstract

There is no previous research that has explored the correlation between compulsive buying and hoarding in the Chinese population. This study aims to determine the relationship between compulsive buying and hoarding in a sample of the Chinese population comprising participants from mainland China (emerging economy) and Hong Kong (developed economy). Self-reported measures consisting of demographic questions, the Chinese version of the Hoarding Rating Scale (CHRS), and Richmond Compulsive Buying Scale-Traditional Chinese (RCBS-TC) were administered to participants. After data collection, common method biases were precluded. The RCBS-TC and CHRS were validated by confirmatory factor analysis and found correlated by Pearson correlation coefficient. The RCBS-TC and CHRS demonstrated satisfactory levels of internal consistency (Cronbach’s α = 0.872 and 0.828, respectively). A three-factor model, including hoarding, obsessive-compulsive, and impulse control disorders, was obtained through Confirmatory Factor Analysis (CFA) with the satisfactory fit for the total sample from Hong Kong and mainland China. A significant correlation was found between RCBS-TC and CHRS (*r* = 0.473). Findings also showed that 14% of the participants exhibited compulsive buying behavior. This study provides sufficient proof of the reliability and validity of RCBS-TC and CHRS. Their relationship was explored based on two sets of samples from different regions in Asia, which contributes more applicability in a cross-cultural context.

## Introduction

Compulsive buying refers to the chronic, repetitive purchasing behavior in response to negative events and/or feelings (Faber and O’Guinn, 1989, 1992). Such behavior is difficult to control and produces adverse consequences such as financial problems, dysfunction of social and daily life, and emotional harm. Ninan et al. (2000) described compulsive buying as “impulsive and/or compulsive buying of unneeded objects” (p. 362) and Black (2001) defined it as “excessive shopping cognitions and buying behavior that leads to distress or impairment” (p. 14). However, the classification of compulsive buying has always been a controversial issue. According to McElroy et al. (1995), compulsive buying is an impulse control disorder that should be classified to a compulsive–impulsive spectrum in the diagnosis. The recurrent and intrusive urges causing stress and anxiety, which are alleviated by carrying out a purchase, can be observed in obsessive-compulsive disorder (OCD). However, others have argued that compulsive buying aimed at triggering euphoria is more like an impulse control disorder (ICD), and not OCD (Christenson et al., 1994). A growing number of researchers claim that compulsive buying should be considered within the dimensions of ICD and OCD because of the overlap of the two disorders (McElroy et al., 1994; Hollander and Allen, 2006). Ridgway et al. (2008) forwarded an expanded conceptualization that compulsive buying is a the tendency of a consumer to be preoccupied with buying, which is revealed through repetitive buying and lack of impulse control over buying.

Previous studies on compulsive buying have mainly been conducted in developed countries or regions (e.g., Faber and O’Guinn, 1992; Koran et al., 2006; Ridgway et al., 2008; Mueller et al., 2009). In recent years, there have been several studies focused on this shopping behavior in emerging countries (Horváth et al., 2013; He et al., 2018). A meta-analytic systematic review of compulsive buying behavior literature revealed that the pooled prevalence of compulsive buying in adult representative samples ranged from 3.4 to 6.9%, although estimates are higher among university students, ranging from 5.9 to 11.5% (Maraz et al., 2016). Thomas et al. (2016) reported that the pooled prevalence of compulsive buying among Emirati female college students was 44.4%, which was the highest reported in all published articles. A study conducted by He et al. (2018) using a Chinese population-based sample found the prevalence to be 29.1%. It has been universally acknowledged that women are more likely to be compulsive buyers (Faber and O’Guinn, 1992; Christenson et al., 1994; McElroy et al., 1994; Dittmar, 2004). Black (2007) argued that the gender differences may be artifactual because women tend to show greater interests in shopping while men like to report that they “collect.”

Just as profound as compulsive buying, hoarding has aroused the interest of psychiatrists in the past decades. Hoarding was first regarded as a measure to cope with shortages and scarcity during the 1970s in the United States (Johnson et al., 1980; Pike, 1981). McKinnon et al. (1985) regarded hoarding as a type of inventory accumulation distinct from other types of inventory accumulation in terms of risk, quantity, and level. Frost and Hartl (1996) defined compulsive hoarding as the “acquisition of possessions which are useless or of limited value; living spaces are sufficiently cluttered so as to preclude activities for which those spaces were designed; significant distress or impairment in functioning is caused by the hoarding” (p. 341). The definition distinguished hoarding from collecting, which is generally viewed as a hobby. A person diagnosed as a hoarder shows the symptoms of clutter and acquisition of items bought and acquired free of costs, such as extra brochures and discarded trash (Shafran and Tallis, 1996; Hartl and Frost, 1999). While there is evidence to support the view that hoarding is a distinct subtype of OCD, the treatments for OCD do not play the same effective role in the case of compulsive hoarding (Black et al., 1998; Mataix-Cols et al., 1999). Nowadays, hoarding has been classified as a psychiatric entity within the category of OCD in the fifth edition of the Diagnostic and Statistical Manual of Mental Disorders-Fifth Edition (DSM-V) [American Psychiatric Association (APA), 2013].

The prevalence of hoarding has been estimated through samples in different contexts. In a German population-based sample, the prevalence of hoarding was around 4.6% without significant age and gender differences (Mueller et al., 2009). A low lifetime prevalence rate of hoarding of 2% among individuals with no mental disorders was reported in the European Study of the Epidemiology of Mental Disorders (Fullana et al., 2010). However, Samuels et al. (2008) found a prevalence of hoarding of 4%, which increased with age and was two times as high in men as women. The prevalence of hoarding was also inversely related to household income (Samuels et al., 2008). New studies are required to determine if hoarding is associated with the different stages of economies (emerging economies and developed economies) since financial insecurity could result in compulsive hoarding.

A common belief is that compulsive buying is associated with hoarding because nearly all hoarders suffer from compulsive buying, while not all compulsive buyers are hoarders (Frost et al., 1998, 2002; Mueller et al., 2007, 2009). In a representative sample of Germans, approximately 61% of the participants classified as hoarders were diagnosed as suffering from compulsive buying. In contrast, 39% of the participants suffering from compulsive buying were reported to be compulsive hoarders (Mueller et al., 2009). In their 2008 research, Mueller et al. found that compulsive buyers with hoarding symptoms reported more severe obsessive-compulsive symptoms and presented with higher psychiatric co-morbidity as compared with non-hoarding subjects. It is important to determine the relationship between compulsive buying and hoarding and differentiating these subgroups of compulsive buyers because specific therapeutic measures might be necessary for compulsive buyers who are also considered hoarders.

Frost and Hartl (1996) considered that acquisition should be incorporated into the definition of compulsive hoarding because compulsive buyers scored high on the Magazine/Newspaper Questionnaire. This questionnaire is an instrument that measures thoughts that occur in a hoarding-related context. Later, Frost et al. (2002) found that compulsive buyers had higher scores in the Compulsive Acquisition Scale (CAS). The acquisition was already considered as part of hoarding (Frost and Hartl, 1996; Frost et al., 1996), which means that those findings are consistent with the suggestion that compulsive buying and hoarding are heavily overlapping phenomena (Frost and Hartl, 1996; Frost et al., 1998). Lo and Harvey (2014) found that compulsive buyers in a sample of Taiwanese were driven by obsessive acquisition and tended to exhibit hoarding behaviors that originated from their compulsive buying. Their British counterparts were only prone to being obsessive in acquiring certain products. Interestingly, Lawrence et al. (2014) suggested that hoarding was an important predictor for compulsive buying.

Compulsive buying and hoarding are associated with OCD (Frost et al., 1998, 2002) and related most closely to impaired mental control, a feature of OCD (Frost et al., 1998). Frost et al. (2002) found that the relationship between compulsive buying and OCD was largely mediated by hoarding. However, the association between compulsive buying and OCD was not prominent because hoarding was underrepresented in the OCD samples. This disparity can be solved by incorporating the OCD dimension in the definition of hoarding or the related questions in the diagnostic protocol while assessing hoarding symptoms (Ball et al., 1996; Frost et al., 2002).

Based on the above discussion, issues in compulsive buying, hoarding, and the relationship between the two concepts need to be addressed. To our knowledge, no studies have been conducted in the Chinese population-based sample from both mainland China and Hong Kong. Therefore, for determining the relationship between compulsive buying and hoarding, it is necessary to carry out a study on compulsive buying and hoarding where the subjects are selected from a Chinese population-based sample.

In the present study, we investigated compulsive buying and hoarding in a Chinese population-based sample using self-administered questionnaires. The purpose of the study was to explore the association between compulsive buying and hoarding based on a sample of Chinese from mainland China and Hong Kong. It could also create an opportunity to explore the relationship between compulsive buying, hoarding, and stages of economic development as Hong Kong is a developed region and mainland China is an emerging economy.

## Methods

### Design

This study employed a cross-sectional, multi-center, and correlational design.

### Sampling and Sample Size Estimation

All data were obtained *via* an online survey. A survey was conducted in mainland China through Sojump, a popular online consumer panel survey company. Sojump developed a database of more than 2.6 million consumers from different cities in China. Participants received points as a reward for participation in the survey, and the points could be redeemed for various gifts. The online survey generated 632 valid responses. In Hong Kong, we created a website questionnaire. The link to the webpage was posted on Facebook and an online forum in Hong Kong. The online site collected 642 valid responses.

### Measurements

The questionnaires consisted of three parts, including demographics, the Chinese Hoarding Rating Scale (CHRS), and Richmond Compulsive Buying Scale-Traditional Chinese (RCBS-TC). The demographic data included gender, age, marital status, education, and income level. Five questions were based on hoarding dimensions, including clutter, difficulty discarding, excessive acquisition, distress, and impairment, and six items that were about compulsive buying were divided into OCD and ICD dimensions.

#### Chinese Hoarding Rating Scale

The Hoarding Rating Scale-Interview (HRS-I) was first developed by Tolin et al. (2010a) as a semi-structured diagnostic interview to fill the blanks in the assessment of hoarding, where only structured self-report questionnaires and direct observations [Saving Inventory-Revised (SI-R), Clutter Image Rating (CIR)] were available. The scale contains five questions, including the proposed dimensions of hoarding, namely, clutter, difficulty discarding, excessive acquisition, distress, and impairment. Each item is measured on a 9-point Likert-type scale from 0 (none) to 8 (extreme) by trained raters based on clinician judgment. A higher score indicated more severe hoarding behaviors. Receiver operating characteristic (ROC) analysis had been conducted by Tolin et al. (2010a) to determine the capability of HRS-I items and total score to discriminate between individuals with and without hoarding. The results proved that 14 was the optimal cut-off score for the total HRS-I score. The HRS-I (interview version) was translated and revised into CHRS (self-reported version). Four items were added to recap the current conditions of stocking to improve the accuracy of self-reporting. Respondents were asked to review the number of items, such as shoes, bags, and t-shirts, stored in their houses in large amounts. CHRS has been used in a large-scale population survey (*N* = 1028) (Li, 2017). Based on the data, the reliability of CHRS was satisfactory (Cronbach’s α = 0.86, corrected item-total correlation coefficients = 0.60–0.74, intraclass correlation coefficient of 2-week test-retest reliability = 0.78) (Liu et al., 2020). The content validity (Content Validity Index (CVI) = 0.80–1.00, ratings from six healthcare and social science experts in compulsive behaviors), face validity (100% comprehensibility from 20 public samples), and construct validity (verified by the exploratory and confirmatory factor analyses) were satisfactory (Liu et al., 2020). CHRS was used to assess the compulsive hoarding behavior in this study.

#### Richmond Compulsive Buying Scale-Traditional Chinese

The Richmond Compulsive Buying Scale-Traditional Chinese (RCBS-TC) (Lam et al., 2018) is the translated version of the Richmond Compulsive Buying Scale (RCBS) (Ridgway et al., 2008). Exploratory and confirmatory factor analyses were conducted to analyze the two-factor structure of the RCBS. Lam et al. (2018) translated the RCBS comprehensively into traditional Chinese (reliability was found sufficient with Cronbach’s α = 0.86, ICC = 0.82) and confirmed the validity [face validity = 100%; content validity = 0.83–1.00 for Item-CVI, 0.97 for Scale-CVI; Confirmatory Factor Analysis (CFA) = 0.99 for Comparative Fit Index (CFI), 0.98 for NFI, 0.99 for IFI, and 0.089 for Root Mean Square Error of Approximation (RMSEA)] of RCBS-TC. The Chinese version of this 6-item self-report instrument uses a 7-point Likert scale. In the original RCBS, the cut-off point of the compulsive buying index to discriminate compulsive buyers from non-compulsive buyers was 25 (Ridgway et al., 2008), whereas He et al. (2018) recommended that using a beginning point of 29 was more suitable with Chinese customers. In addition to the RCBS-TC, we added one question on the frequency of buying the things, as shown in items 11 and 12 for data analysis.

### Data Analysis

We compared the participants who reported hoarding and compulsive buying behaviors with the participants who did not, from Hong Kong and mainland China. The analysis, including descriptive factors (e.g., percentages, means, and SDs) and inferential statistics (e.g., *t*-test, chi-square, and Cronbach’s α), was conducted with SPSS software, version 22.0 for Windows. The Linear Structural Relations software, version 8.70 for Windows was used for the CFA. Common method bias was checked to confirm the distinct construct of two scales in three dimensions (Podsakoff et al., 2003).

## Results

### Descriptive and Sociodemographic Results

A total of 1,274 valid samples comprised 664 women and 610 men, taking up 47.9 and 52.1% shares, respectively. However, since some of the original options regarding age, marital status, education, and income level were lacking in sufficient samples, we combined several options. Marital status was re-grouped into two categories in which the marital status of all the participants fell in the option of single, including being single again after marriage (*N* = 629, 49.4%) and married containing cohabiting (*N* = 645, 50.6%). Around 46.2% (*N* = 589) of the participants had a bachelor’s or higher degree and 47.9% (*N* = 610) were under the age of 30. Although the level of income in mainland China is generally lower than that in Hong Kong, the sample size in the original interval of monthly income, namely, lower than HK$3000, HK$3001–5000, and HK$5001–10,000, was insufficient to determine the income differences between participants from Hong Kong and mainland China. As a result, these three options were merged into one, and we found that the monthly income of 55.8% (*N* = 710) of the participants was lower than HK$10,000.

As shown in Table 1, the participants from Hong Kong (*N* = 642) and mainland China (*N* = 632) differed in age, marital status, education, and income. On average, the participants from mainland China were younger compared to participants from Hong Kong, but the latter were wealthier as expected. The education level among mainland Chinese participants was not balanced, with more participants having degrees but a lesser number holding high school certificates. A huge disparity in gender was not seen.

**TABLE 1:** Descriptive statistics of samples of Hong Kong, Mainland China, and total (*N* = 1274).

**Descriptive statistics**	**Data source**			***p*-value**
	**Hong Kong (*n* = 642)**	**Mainland China (*n* = 632)**	**Total**	
**Gender**				0.459
Male	314 (48.9%)	296 (46.8%)	610 (47.9%)	
Female	328 (51.1%)	336 (53.1%)	664 (52.1%)	
**Marital status**				< 0.001*
Single	394 (61.4%)	235 (37.2%)	629 (49.3%)	
Married	248 (38.6%)	397 (62.8%)	645 (50.7%)	
**Education**				< 0.001*
Secondary or below	212 (33.0%)	247 (39.0%)	459 (36.0%)	
Higher education	168 (26.2%)	58 (9.2%)	226 (17.7%)	
Degree or above	262 (40.8%)	327 (51.7%)	589 (46.2%)	
**Age**				< 0.001*
18–29	317 (49.4%)	293 (46.4%)	610 (47.9%)	
30–39	125 (19.5%)	246 (38.9%)	371 (29.1%)	
40–49	67 (10.4%)	66 (10.4%)	133 (10.4%)	
50 or above	133 (20.7%)	27 (4.3%)	160 (12.6%)	
**Income monthly (US$)^a^**				< 0.001*
<1,282	190 (29.6%)	520 (82.3%)	710 (55.8%)	
1,283–2,564	217 (33.8%)	74 (12.2%)	291 (22.9%)	
2,565 or above	234 (36.4%)	38 (6.0%)	272 (21.4%)	

*p < 0.001.

*^*a*^Only 1273 valid samples.*

### Common Method Bias Analysis

Common method bias (also named as common method variance), which is usually caused by single measurement and data from the same source, can be ascribed to the measurement method rather than to the constructs (Bagozzi and Yi, 1991; Podsakoff et al., 2003). Particularly, many previous studies did not take common method bias into account but reported the mild to the medium association between several self-reported symptoms under the field of obsessive-compulsive disorders (Frost et al., 1998, 2002; Mueller et al., 2007, 2009). There is a paucity of studies to address such a methodological gap.

Common method bias should be reviewed prior to the main study (Bagozzi and Yi, 1991; Podsakoff et al., 2003). In our questionnaire, the original semi-structured hoarding rating scale was transformed to a self-reported measure that was distributed together with self-reported RCBS, which easily could give rise to common method bias (Voorhees et al., 2016). With that, in this study, common method bias of one-factor structure of CHRS as well as two-factor structure of RCBS-TC was evaluated through comparing three set of CFA models. This method can be considered as using the correlated uniqueness model, a technique recommended by Podsakoff et al. (2003) as a statistical remedy for common method biases. The correlated uniqueness model can account for method effects by allowing the error terms of variables, which were measured by the same method to be correlated. The primary advantage of the correlated uniqueness model is that the effects of multiple method biases can be examined at the same time. As the number of latent variables increased gradually, the model yielded more satisfactory goodness-of-fit indices (as shown in Table 2), thereby suggesting that common method bias did not pose a significant problem.

**TABLE 2:** Goodness-of-fit indices within hoarding, OCD and ICD dimensions.

**Dimensions**	**Goodness-of-fit indices**							
	***X* ^2^**	**df**	**RMSEA**	**NNFI**	**CFI**	**SRMR**	**MAIC**	**SAIC**
**One dimension^a^**								
Total	2416.38	44	0.206	0.82	0.86	0.110	2460.38	132
Hong Kong	1457.46	44	0.224	0.84	0.87	0.110	1501.56	132
Mainland China	1179.51	44	0.202	0.76	0.81	0.120	1223.51	132
**Two dimensions^b^**								
Total	442.44	43	0.085	0.96	0.97	0.039	488.44	132
Hong Kong	501.97	43	0.129	0.94	0.95	0.056	547.97	132
Mainland China	139.35	43	0.060	0.97	0.98	0.040	185.35	132
**Three dimensions^c^**								
Total	299.90	41	0.070	0.97	0.98	0.036	349.90	132
Hong Kong	329.30	41	0.105	0.96	0.97	0.051	379.30	132
Mainland China	106.16	41	0.050	0.98	0.98	0.039	156.16	132

*^*a*^All observed variables.*

*^*b*^Hoarding and CB dimensions.*

*^*c*^Hoarding, OCD and ICD dimensions.*

#### Reliability and Validity

The Cronbach’s α of RCBS-TC was 0.872, with corrected item-total correlation coefficients of 0.592–0.763. Both coefficients indicated that the scale had satisfactory internal consistency. The Cronbach’s α of CHRS was 0.828 with corrected item-total correlation coefficients of 0.560–0.712, which indicates satisfactory internal consistency.

Starting with one latent variable, CFA was conducted three times with one latent variable added each time. Table 2 shows the CFA results of the total sample and the samples in Hong Kong and mainland China separately. In this study, CFA, which used the three-factor model containing hoarding, obsessive–compulsive, and impulse-control dimensions, demonstrated the most acceptable fit for the three samples (total sample: *X*^2^ = 299.9, *p* < 0.1, Non-Normed Fit Index (NNFI) = 0.97, CFI = 0.98, Standardized Root Mean square Residual (SRMR) = 0.036, RMSEA = 0.07; Hong Kong sample: *X*^2^ = 329.3, *p* < 0.1, NNFI = 0.96, CFI = 0.97, SRMR = 0.051, RMSEA = 0.105; mainland Chinese sample: *X*^2^ = 106.16, *p* < 0.1, NNFI = 0.98, CFI = 0.98, SRMR = 0.039, RMSEA = 0.05).

All five items of CHRS and six items of RCBS-TC have a factor loading at or above 0.5 (as shown in Table 3). The obsessive-compulsive and impulse-control dimensions were highly correlated in the model of three samples (Total and mainland Chinese samples: ρ = 0.87, Hong Kong sample: ρ = 0.85; *p* < 0.1).

**TABLE 3:** Standardized item loading of samples of total, Hong Kong, Mainland China within hoarding, OCD and ICD dimensions.

**Dimensions**	**Standardized item loading**		
	**Total sample**	**Hong Kong sample**	**Mainland Chinese sample**
**Hoarding dimension**			
Clutter	0.61	0.64	0.61
Difficulty discarding	0.60	0.65	0.62
Acquisition	0.63	0.69	0.56
Distress	0.83	0.85	0.80
Impairment	0.82	0.86	0.78
**OCD dimension**			
My closet has unopened shopping bags in it	0.63	0.68	0.53
Others might consider me a “shopaholic”	0.88	0.90	0.86
Much of my life centers around buying things	0.80	0.83	0.77
**ICD dimension**			
I consider myself an impulse purchaser	0.82	0.87	0.75
I buy things I don’t need	0.76	0.80	0.73
I buy things I did not plan to buy	0.68	0.81	0.50

*The *p*-value of the listed standardized item loadings is less than 0.05.*

The average variance extracted (AVE) of the hoarding dimension was 0.4981, which was lower than 0.5, but maybe acceptable because composite reliability (0.8292) was higher than 0.6, and hence, the convergent validity of the construct was still adequate (Huang et al., 2013). The AVE and composite reliability of the Hong Kong sample were higher than the mainland Chinese sample in terms of the three dimensions (Table 4).

**TABLE 4:** Average variance extracted and composite reliability of samples of Total, Hong Kong, and Mainland China within hoarding, OCD and ICD dimensions.

**Dimensions**	**Total sample**		**Hong Kong sample**		**Mainland China sample**	
	**CR**	**AVE**	**CR**	**AVE**	**CR**	**AVE**
Hoarding dimension	0.8292	0.4981	0.8593	0.5541	0.8090	0.4637
OCD dimension	0.8178	0.6038	0.8483	0.6538	0.7709	0.5378
ICD dimension	0.7987	0.5708	0.8666	0.6843	0.7032	0.4485

### Correlation Between Hoarding and Compulsive Buying

Pearson correlations were used between RS-TC and RCBS-TC to explore the relationship between compulsive buying and hoarding. The CHRS scores correlated significantly with the RCBS-TC scores but were not very strong (*r* = 0.473, *p* < 0.001). Considering the interrelations among the subscales of compulsive buying and hoarding, unlike the findings of Mueller et al. (2009), the strongest correlation was found between the impairment subscale and RCBS-TC (*r* = 0.430, *p* < 0.01), but not the acquisition subscale. The weakest association was observed between the difficulty discarding subscale and RCBS-TC (*r* = 0.293, *p* < 0.01), which was consistent with the results of Mueller’s research, but weaker.

## Discussion

The aim of this research was to explore the relationship between compulsive buying and hoarding. Our study was the first to compare compulsive buying and hoarding behaviors considering large samples from Hong Kong and mainland China. At the same time, our study contributed more generalizability to the findings in a cross-cultural context.

Common method bias was the first to be precluded in our research. Internal consistency assessment suggested that the RCBS-TC and CHRS exhibited satisfactory internal consistency with Cronbach’s α of 0.872 and 0.828, respectively. CFA indicated that a first-order three-factor model was more acceptable in the Chinese population when compulsive buying and hoarding behaviors were measured together. With one factor of hoarding and two factors (OCD and ICD) of compulsive buying, the CFA results were consistent with the findings of Ridgway et al. (2008) and Tolin et al. (2010b).

Significant correlations were observed between the measures of hoarding and compulsive buying. The associations between the two scales and their subscales were weaker than what was found by preceding scholars (Tolin et al., 2010b). In the present research, the impairment item (subscale of CHRS) had the strongest correlation with RCBS-TC, which was due in part to the society undergoing rapid development. The reform and opening-up policies brought more opportunities to the Chinese, but simultaneously caused “urban disease,” such as excessive pressure and anxiety. In 2012, the annual Work-Life Balance Index released by Regus, the world’s largest provider of flexible workspace solutions, pointed out that the pressure on mainland Chinese workers ranked first in the world. Among the 16,000 professionals in 80 countries and regions around the world, 75 and 55% of professionals, respectively, from mainland China and Hong Kong thought the pressure was higher than that in 2011. The figures showed that the top three reasons for pressure were work, personal finances, and pressure from leaders (Regus, 2012). According to Tolin et al. (2010a), hoarding symptoms were linked closely to stressful and dramatic life events, and thus, Chinese respondents scored highest in the impairment subscale because they were burdened with greater pressures in life. However, in the research by Mueller et al. (2009), the acquisition subscale had the strongest correlation with RCBS-TC while the difficulty discarding subscale had the weakest association with RCBS-TC, which has also been observed in prior and present studies.

Limitations should also be considered. On one hand, the sample we utilized had not been identified as clinically compulsive buyers and hoarders or found seeking treatment before we conducted the research. Hence, this limitation could affect the prevalence rate of compulsive buying and hoarding, and we also lost the opportunity to find out the appropriate cut-off point for Chinese hoarders. On the other hand, the use of self-report instruments could lead to response biases despite CHRS, which was adapted from the original semi-structured instruments and RCBS-TC, which was well validated.

To sum up, the present research is the first to investigate the association between compulsive buying and hoarding in a Chinese population-based sample with participants from Hong Kong (developed economy) and mainland China (developing economy). Through a comparison of the compulsive buying and hoarding behaviors of two sets of participants, we can further understand their discrepancies, and targeted treatments can be developed in the future.

## Data Availability Statement

The original contributions presented in the study are included in the article/supplementary material, further inquiries can be directed to the corresponding author/s.

## Ethics Statement

The study procedures were carried out in accordance with the Declaration of Helsinki. The Institutional Review Board of the University Research Centre, The Open University of Hong Kong (HE20Jul2017-S&T2017/01). The patients/participants provided their written informed consent to participate in this study.

## Author Contributions

HH and SL were involved in the development of the idea, project supervision, and data collection. HH, MZ, and SL carried out data analysis, literature review, draft of the manuscript, and review of the manuscript. All authors reviewed and approved the final version of the manuscript.

## Conflict of Interest

The authors declare that the research was conducted in the absence of any commercial or financial relationships that could be construed as a potential conflict of interest.

## Publisher’s Note

All claims expressed in this article are solely those of the authors and do not necessarily represent those of their affiliated organizations, or those of the publisher, the editors and the reviewers. Any product that may be evaluated in this article, or claim that may be made by its manufacturer, is not guaranteed or endorsed by the publisher.
